# Philadelphia County Medical Society

**Published:** 1890-05

**Authors:** 


### PHILADELPHIA COUNTY MEDICAL SOCIETY.

Stated meeting February 26, 1890.
The president, Dr. W. W. KEEN, in the chair.

### A SYMPOSIUM OF ABDOMINAL SURGERY.

FIRST PAPER.

### OPERATIVE METHODS AS ILLUSTRATED BY THE HISTORY OF OVARIOTOMY IN AMERICA.

BY LEWIS S. MCMURTRY, M. D.,
Louisville, Ky.
(By invitation.)

THE history of ovariotomy is the history of abdominal surgery. For many years after the establishment of this operation, abdominal surgery was limited to the removal of ovarian tumors. The knowledge and experience gained thereby led to other procedures, until the present proud position of peritoneal surgery was attained. Beginning in America in 1809, it has been persistently and zealously cultivated by American surgeons up to the present time. A retrospect of the origin, early history, struggles, and triumphs of this heroic branch of surgery is replete with instruction. That its pioneers were subjected to criticism and abuse, leading oftentimes to professional ostracism; that they were treated with incredulity, and charged with legalized murder; and throughout all this misrepresentation and persecution that they pursued the stern line of duty, are circumstances which make this the most thrilling chapter in the history of American surgery. It is the purpose of this paper to deal with methods rather than men, and to endeavor to deduce from the record some lessons bearing upon the practice of the present time.

In December, 1809, Dr. Ephraim McDowell,[1] at his home in Dan-

1. See frontispiece for an illustration of the home of McDowell, where his operations were performed.

ville, Kentucky, performed the first ovariotomy. The writer of this sketch was born and reared near this town, educated there, and resided there until recently. Having known since my boyhood the immediate family and former patients of Dr. McDowell, I have had exceptional opportunities to inform myself as to the operator and his work. In 1793 and 1794 McDowell was a student of medicine in Edinburg, where he was a pupil of John Bell. From this eminent teacher he learned the nature of ovarian tumors, and was informed of the perils which would be attached to any attempt at their removal. He knew that no surgeon, however great his skill, had ever attempted such a bold and perilous procedure. Fifteen years after his return to America, after attaining wide-spread celebrity as a surgeon, especially as a lithotomist, he performed his first ovariotomy. The operation, of course, was executed without an anesthetic. The incision was made to the left of the median line, about three inches external to the border of the rectus muscle, and was nine inches in length. After opening the peritoneum, he first tied the pedicle with a strong ligature, and then cut open the tumor and removed its contents. He then divided the pedicle and removed the sac. As soon as the incision was made into the abdomen, he states, the intestines rushed out upon the table, and were not replaced until the operation was completed, which, he adds, occupied twenty-five minutes. He then turned the patient upon the left side to allow all fluids to escape. He closed the incision with interrupted sutures, and brought out the ligature attached to the pedicle at the lower angle of the wound. Adhesive strips and a bandage completed the dressing; the patient was put to bed, and he prescribed "a strict observance of the antiphlogistic regimen." At the end ot twenty-five days the patient returned home in good health.

The special features of McDowell's technique worthy of note are: (1) The incision was made through the muscular layers of the abdominal wall, three inches external to the rectus muscle. (2) The cyst was not evacuated until after the pedicle was ligated. (3) An effort was made to cleanse the peritoneum of fluids. (4) Drainage was sought by bringing the ligatures out at the lower angle of the wound. (5) The operation occupied only twenty-five minutes, expedition being more hastened, doubtless, by the absence of an anesthetic than otherwise.

McDowell operated thirteen times, with the result of eight recoveries and five deaths. In his report of his second case he uses the expression: "I laid her side open," as in the former case. In his third case he adopted the median incision, saying in his report of this case: "I changed my place of opening to the linea alba."

Dr. McDowell was a man of splendid presence and aristocratic bearing. He was quite a "dressy" man, neat in his appearance and cleanly in his person and habits. That McDowell appreciated the responsibility he had incurred, and was aware that he was establishing a great operation, destined to add lustre to the science and art of surgery, is apparent in the following extract from his reply to a Dr. Henderson, who had criticised McDowell's report for its want of "particular detail." Replying through the columns of the *Eclectic Repertory and Analytical Review*, of Philadelphia, McDowell says:

> I thought my statement sufficiently explicit to warrant any surgeon's performing the operation when necessary, without hazarding the odium of making an experiment, and I think my description of the mode of operating, and of the anatomy of the parts concerned, clear enough to enable any good anatomist, possessing the judgment requisite for a surgeon, to operate with safety. I hope no operator of any other description may ever attempt it. It is my most ardent wish that this operation may remain to the mechanical surgeon forever incomprehensible.

This quotation stamps McDowell as a surgeon of broad and elevated views, fully aware that he had opened a new and rich domain in surgery.

McDowell first operated in 1809, and in July, 1821, Dr. Nathan Smith, Professor of Surgery in Yale College, performed ovariotomy at Norwich, Connecticut. Dr. Smith had never heard of McDowell's work, and operated in an entirely original way. The incision was made in the median line, and was three inches in length. He states that he waited until the blood ceased flowing before he opened the peritoneum. The omentum being adherent, he tied it away with ligatures made from a kid glove. The pedicle was also tied with the same material, the ligatures cut short, the pedicle dropped, and the incision closed. The patient made an uninterrupted recovery, and sat up and walked at the end of three weeks. Dr. Smith undertook another operation for ovarian tumor, but desisted on account of adhesions, and closed the incision. The patient recovered from the operation. Notable features of his technique were: (1) the short incision; (2) protection of the peritoneum from the blood of the incision; (3) ligature of omental adhesions; (4) ligature of pedicle, ligature cut short, and pedicle dropped; (5) animal ligatures.

Dr. Alban G. Smith, of Danville, Kentucky, was the third to perform ovariotomy. He assisted McDowell in several of his operations, and operated on May 23, 1823. He reported the operation in the *North American Medical and Surgical Journal*, January, 1826. He followed McDowell's method in operating, and brought the ligature out through the lower angle of the wound. His patient recovered. That surgical opinion and practice often travel in a circle is aptly illustrated by the fact that he administered a decoction of senna to the patient the night after the operation, producing free evacuations the next day, and several the day after. Peaslee mentions this to illustrate the crude methods of early days. We all know now that such medication is based on sound principles, and Dr. Alban G. Smith would, if living, find himself thoroughly *en rapport* with the most advanced methods of the day.

McDowell died in 1830. Nathan Smith did not press his work further after his second operation, which was only an exploratory incision. From this period until 1843 the operation lapsed into obscurity, and was practically abandoned by the profession. A few brave surgeons would occasionally attempt it only to abandon their purpose when adhesions were encountered. Among these were Trowbridge, of New York; Mussey, of Dartmouth; and a few others. In 1829, Rogers, of New York, operated successfully, adopting the method of treating the pedicle which Nathan Smith had practised. Billinger, of Charleston, S. C., operated successfully in 1835, trying the pedicle with animal ligatures, cut short, and pedicle dropped, after Nathan Smith's method. From 1835 until 1843 there were absolutely no cases of ovariotomy in this country. In the latter year, Dr. John L. Atlee, of Lancaster, Pa., and Dr. Alexander Dunlap, of Springfield, Ohio, began their work, joined by Dr. Washington L. Atlee, of Philadelphia, the following year, and revived the operation in America.

In England, Charles Clay did his first operation in 1842. Mr. Spencer Wells began to operate in 1858. The status of the operation in Great Britain at the time the Atlees and Dunlap began their work may be inferred from the fact that the first successful ovariotomy in a London hospital occurred in 1846, Mr. Cesar Hawkins being the operator, three years after the revival of the operation by the Atlees and Dunlap. J. Taylor Bradford, of Kentucky, entered upon his series of thirty operations, with twenty-seven recoveries in 1853; and Gilman Kimball, of Lowell, Mass., joined his colleagues in the great work in 1855. Peaslee states that the operation did not secure a permanent footing in the United States until 1855, and then only in the country. The surgeons of the cities, as a rule, opposed it for some years afterward. Ten years later, 1865, the operation was generally accepted. Operative surgery received a great impetus in the discovery of anesthesia.

No one can read Dr. John L. Atlee's report of his first case, which was the first double ovariotomy ever performed, without recognizing the courage, skill, and sagacity of a master in surgery. He operated with rapidity; he carefully arrested all bleeding; he cleansed the peritoneum, and used silk ligatures. He transfixed the pedicle with a double silk ligature, and tied each half with half the ligature. He adopted McDowell's method, and brought the ligatures out through the lower extremity of the wound to secure drainage. Dr. Washington L. Atlee performed his first operation in March, 1844. It was unsuccessful. His next was done five years later, and was successful. He adopted the methods of his brother in operating. Dunlap treated the pedicle after McDowell's method. Dr. E. R. Peaslee began his series of ovariotomies in 1850, and Sims, Thomas, and Emmet, a few years afterward joined in the work. In October, 1871, Peaslee had collected 739 ovariotomies performed in this country, with successful results amounting to about sixty-eight per cent.

In 1858 Mr. Jonathan Hutchinson, of London, performed two ovariotomies, and devised the clamp for fixing the pedicle externally. This method was adopted and modified by W. L. Atlee, who led all American operators of his day in the number of his cases. The clamp came into general use, and had the confidence of ovariotomists generally until a recent period. For many years the method of securing the pedicle was considered the pivotal point in the operative technique, and the ingenuity of operators was expended on this feature of the operation. Under the leadership of Spencer Wells in England and Atlee in the United States, the clamp superseded other methods.

The causes of death after ovariotomy were enumerated at this same period (1873) by Peaslee, as follows, his deductions being based upon post-mortem examination of fifty cases. The causes are given in the order of frequency: Peritonitis, septicemia, shock, exhaustion, hemorrhage, strangulation of intestine, diarrhea, erysipelas, tetanus, and ulceration through the bladder. Based upon this view of the pathology, peritonitis was regarded as the greatest danger to which a patient was subjected after ovariotomy, and opium in heroic and repeated doses, in accordance with the accepted therapeutics of that day, was the conspicuous feature of the after-treatment. This treatment was universally accepted by the profession. The turning point in the history of ovariotomy in America dates from the correction of this error in pathology. When it became known that the greatest peril of a patient during and after ovariotomy is septicemia, and that the peritonitis hitherto attributed to traumatism was in reality due to septic infection, this department of surgery entered upon an advance which in grandeur and brilliancy has no parallel in the history of our art. This great fact was heralded in America by Marion Sims and met with universal acceptation. Sims instituted a method of drainage after ovariotomy by puncturing Douglas' cul-de-sac and passing a tube into the vagina. This was soon abandoned, though it is now practised by Martin, of Berlin.

With the acceptance of the fact that the supreme danger after ovariotomy is in septic infection, a new era in ovariotomy began in America. Synchronous with the adoption of operative methods and after-treatment based upon this knowledge the clamp was abandoned, and the intra-peritoneal treatment of the pedicle was restored. The most important single contribution made to pelvic and abdominal surgery is the system of surgical cleanliness instituted by Lister. Burdened with the spray, pads, macintosh, and chemical solutions at first, it has been simplified and stripped of its dangers and its encumbrances, and found to consist, in its essence, of absolute cleanliness. With the application of this principle—the outgrowth in practical surgery of the researches of Pasteur, Lister, Koch, Watson-Cheyne, and others, upon the nature of fermentation, putrefaction, suppuration, and septic infection—results have been obtained of which our predecessors scarcely dreamed. From ovariotomy the work extended to other organs within the peritoneal envelope, until all have been included.

When Marion Sims, a few years ago, confidently predicted that the time was near at hand when gun-shot wounds of the abdomen would be treated by abdominal section and suture of the wounded viscera, it was considered by many as the dream of an erratic man of genius. Already a number of successful cases have been recorded in America. Ovariotomy has within a few years grown from an operation with desperate mortality to be the most successful major operation in surgery. From a last resort, attempted by a few bold spirits in the profession, it is now done habitually by surgeons in every State of the Union. Along with the extension of surgical treatment to the other organs invested by the peritoneum has come a greater perfection in operating, so that the work of the best operators has a completeness and finish approaching the ideal in surgical art.

Time will not permit even an enumeration of the additions contributed in America to modern ovariotomy and the valuable contributions to the surgery of the intestines and other abdominal organs, all of which is the outgrowth of the work inaugurated by McDowell.

In conclusion I will venture to direct attention to one important lesson to be deduced from the study of the history of ovariotomy in the country of its birth. We have seen from what has preceded that the operation grew to its grand proportions in the hands of a few. All who have labored in this field of surgery realize how difficult is diagnosis even with experience; how exacting the technique; how varied the work in apparently similar cases; and how fatal an error. Of all departments of surgical practice none requires such rigid and prolonged apprenticeship for successful results. No surgeon can rely upon simple cases. What is most needed to maintain the high standard fixed for abdominal surgery by its founders and its eminent practitioners is men in every community especially trained and equipped for this special work.

---

SECOND PAPER.

### DRAINAGE IN ABDOMINAL AND PELVIC SURGERY.

BY JOSEPH PRICE, M. D.,

Physician to the Preston Retreat; Surgeon to the Gynecean Hospital, etc.

THE absolute necessity of drainage in abdominal surgery has led to a careful estimate of its value in all surgery. Wherever there is serous effusion or leakage of any kind in the line of incision or in the cavity caused by the removal of any neoplasm, the cicatrization of the wound is interfered with, if not by the nature of the fluid, by the mechanical pressure exerted by that fluid under tension. In the pelvis the presence of fluid as a pathological accumulation at the seat of removal, involves a nidus of remaining unhealthy tissue, where physiological or healthy digestion is impossible. Illustrative of this fact I have the following specimens: In the first there is a pyosalpinx with an appendicitis and cecal ulcer. The cecal ulcer necessitated the stitching of the bowel, while the appendix stump was inverted and closed peritoneally. The pus-tube was universally adherent, and in its enucleation débris of unhealthy tissue was necessarily left to a greater or less extent. The value of drainage is here apparent. In the second case the condition was analogous, save that there were present double pus-tubes with an ovarian abscess.

The third specimen is that of an extra-uterine pregnancy with rupture, recurring hemorrhages, and general peritonitis. The abdomen was full of blood. In a fourth specimen there is a small adherent cyst, with a pus-tube on the opposite side.

The group of cases were operated on in the last ten days, and all the patients were suffering from extreme exhaustion, from loss of blood, and the presence of pus. In all these cases the trouble is universally of long standing, where all imaginable complications existed. Foremost among these complications were the bowel adhesions. In one week I was compelled to stitch the intestine five times. In this connection the care required in dealing with diseased serous surfaces claims especial attention. Not only must the primary lesion of the intestine be intelligently dealt with, but the secondary disease incident to its pathological adhesions, and the ragged remains incident to its separation must also receive extreme attention. In no way can they be rendered inoffensive, save by drainage. In speaking of remaining débris, it may be well to insist upon a distinction between a remainder that is absolutely unavoidable, and that which occurs through timidity, inexperience, or gross carelessness. Such remainders as these are nothing more than criminal. When I find them or hear of them, I know one of two or three things has caused them. When I hear of a patient's being operated upon by the same man two or three times, I estimate the operator's ability or conscience accordingly. All the antiseptics, whether in bottles or consciences, will not make careless surgery give good results, or free the patient from the dangers of repeated operations. In four cases which have lately come under my notice, three of the patients died of operations repeated not less than three times. Flesh and blood will stand about so much. If the tension is too great, the harp-string will strain and snap, be he saint or sinner that plays on it. Necessary remainders are those produced by adhesions in parts not removable, save at a risk greater than is involved by leaving them. Such remainders as these should be trimmed to the utmost. If they are on the bowel, and their presence threatens too much the welfare of the patient, intestinal anastomosis or bowel resection may be resorted to.

Mechanical treatment of all bleeding points need not here be discussed. After all major leakage has been controlled, the hot drenching of the abdominal or pelvic cavity must be resorted to, for the removal of débris, shreds, and clots. After this, even sponge or lint packing may be found necessary. The insertion of the drainage-tube down to the lowest involved locality must then be made. Care must be taken that the tube makes no injurious pressure upon either the diseased or healthy portion of the intestine. In either case it will be fatal to uninterrupted recovery, and is likely to produce fistula. The use of large drainage-tubes, either in abdominal or general surgery, is to be deprecated as unnecessarily impeding healing and encouraging suppuration. A small tube, with a caliber sufficient to insure its not being obstructed by escaping fluids, when somewhat viscid, is all that is called for.

The failure of the earlier operators to appreciate the cause of death in their more complex cases, was, at the bottom, really the cause of that critical period of ovariotomy, when the congestive chills of dread made surgeons tremble and hesitate at deciding for or against the operation. In the earlier post-mortems of patients operated upon, the pelvis was found full of serum and clots. Had the fear of this abdominal sanctuary not been by tradition so dreadful, that it was thought rasher to open it to a drainage-tube than to close it to gallons of fluid and pounds of clot, the tale would have been different and cavilers sooner silenced.

Purulent cases, where a nidus of infection could not by any means be primarily removed, would have yielded to drainage, while they died of turpentine and poultices, that made the belly warm, to hasten the infection. The same is true of all inflammatory products arising from any source whatever. This is a point well illustrated by two cases of puerperal peritonitis I lately operated upon. They had puerperal fever of five weeks standing, with high temperature and general collapse, and were full of pus to the umbilicus. There were huge ovarian abscesses, with bowel adhesions. The tubes and ovaries were removed and the bowel carefully stitched. The abdomens were flooded with hot water twice, which did more to bring up the pulse than anything else. The result is typical—complete recovery without bad symptoms.

It must be insisted upon that the mortality of this work would be so great without drainage, that the cases would have to be left to their fate by the surgeons in simple self-defence. Operators who will not recognize this as proven beyond doubt, will still go on having mortalities that they cannot explain. Their cases, while not worse than others, will suffer by comparison, and their bad results be apologized for.

While operation in all these cases is to be insisted upon, we will constantly see others where typical surgery is impossible; the patients are too far gone to allow prolonged operation. In such cases the immediate cause of the dangerous condition, if pus, is to be eliminated by drenching and drainage, and after the patient has rallied, some days later, the operation may be completed. Too much surgery in extremely debilitated patients will kill just as surely as none at all, as I have long since insisted upon and practised with gratifying results.

---

THIRD PAPER.

### THE TREATMENT OF CERTAIN FORMS OF GANGRENE OF THE INTESTINE.

BY CHARLES B. PENROSE, M.D., PH.D.,

Surgeon to the Gynecean Hospital; Out-Patient Surgeon to the Pennsylvania Hospital.

I PROPOSE to consider briefly here the treatment of the degenerated or gangrenous condition of the intestine which is frequently met with in cases of pelvic or abdominal suppuration. In these cases the intestinal coats are destroyed by direct extension of the inflammation. The condition is common in tubal or pelvic abscess and in the perityphlitic abscess of appendicitis. In such cases we frequently find the abscess cavity walled in by adherent loops of intestine; the intestinal coats being in contact with the purulent accumulation and presenting on their surface one or more cheesy, degenerated spots, of a purple or black color, and from one-half to two inches in diameter. These spots often have in their centers perforations, generally of small caliber, but sufficiently large to permit the escape of pus by the bowel, and often of escape of feces into the abscess cavity. A similar gangrenous condition of the intestine, with or without perforation, is sometimes present in the pelvic inflammatory troubles of women, when the Fallopian tube effects adhesion by its fimbriated end to the sigmoid flexure or rectum, and may discharge its contents through this channel.

Another class of cases in which there is an analogous degenerated condition of intestine and to which the following remarks with regard to treatment also apply, is found in intestinal obstruction, when the pressure upon the intestinal walls from within produces ulceration beginning in the mucous membrane and often extending through all the coats. Such ulceration has not, in my experience, been accompanied by the same amount of inflammatory infiltration and thickening of the diseased areas as occurs when the destructive process is from without inward.

The condition of the patients in whom disease of this character occurs is generally such that they cannot withstand a prolonged or serious operation. Excision of the degenerated patches or resection of the intestine is in most cases out of the question. Evacuation of the abscess, removal of the exciting cause, and adjustment of the abdominal contents in positions most favorable for recovery, are the only steps to be considered. I will, therefore, leave all ideal surgical operations out of the discussion, and consider only those cases in which ideal surgery is impracticable. I will not discuss the best methods of removing the diseased portion of intestine, but the safest way in which it can be left in the abdomen. Such a discussion is of value, because every surgeon must realize the impossibility of performing an ideal operation in many cases of abdominal surgery. Certain rules, theoretical or deduced from experiments on the healthy intestine of the lower animals, are laid down to be followed in the various abdominal operations on man, often without sufficient consideration of the pathological changes and distortions which may be caused by the disease that demands the operation. The determination of the extent to which one can go toward the ideal operation, without sacrificing any of the patient's chances of life to the surgeon's wish for mechanical perfection, distinguishes the true surgeon from the surgical mechanic. And to attain this end the surgeon must not only be familiar with the ideal operations possible in favorable cases, but also with the recuperative powers, and the best means to favor recuperation of diseased or injured structures in complicated cases, where ideal surgery is impossible. In the disease, for instance, which is under consideration, the operator recognizes the danger of immediate, or not very remote, fecal extravasation if the degenerative patches of intestine are not removed, or if the perforations or fistulous openings are not firmly closed, and yet he realizes that the patient may not withstand the ideal operations necessary for the accomplishment of this object. It is then that a knowledge of how much can be safely left untouched, and the best way of so leaving it, is most desirable.

When diseased intestine, such as we are considering, is left in the abdomen, our object should be :

1. To place it in a position which will tend most to favor its restoration to health.

2. To defer as long as possible the separation of the slough and the escape of feces—if such an event is inevitable ; or if a fistulous opening already exists, to defer the escape of feces from this opening until preservative adhesions have taken place.

3. To place the intestine in a position most favorable for the spontaneous closure of a fistula, and most accessible for a subsequent operation.

4. To protect the sound peritoneum from infection from the diseased areas.

One of the most important points in the fulfillment of these indications is the adjustment of the diseased portion of intestine in a straight line, with the gangrenous patch facing the belly wall and in the immediate neighborhood of the abdominal incision. The straight position favors the easy passage of feces past the gangrenous area, and it not only tends to prevent escape of feces, by reducing to a minimum the pressure from within, but it aids, more than anything else, the spontaneous closure of the fistula should one result. The easiest fistula to close is one situated on the side of a straight intestinal tube; the most difficult is one at the apex of a sharp flexure.

If the gangrenous spots face the abdominal wall, the force of gravity will aid not only in preventing or delaying fecal extravasation, but also in closing the fistulous opening. The value of gravity as an agent in the closure of a fistula is well illustrated by a case reported by Sir Henry Thompson, where an old urinary fistula, which opened into the rectum, was cured in a few weeks by making the patient pass his water while lying on his face. By placing the diseased portion of intestine near the abdominal incision, we make it most accessible for drainage, we avoid the risk of a deep fistula, and we have it in a position easy for subsequent operation.

When fistulous openings exist in these gangrenous patches they are generally of small size—often so small that the escape of feces and gas takes place only under pressure. In cases originating in pelvic or abdominal suppuration, the destruction of tissues proceeds from the outer to the inner coats of the intestine, and it generally happens that though pus may escape readily from the abscess into the bowel, yet the intestinal contents find a much less free exit on account of a valve-like closure of the opening. Some of these fistulous openings are so small that, existing as they do in the center of a thickened diseased mass, they cease to be patulous when the intestine is freed from its adhesions, and are often not visible at the time of operation, their presence being only proved by the previous or subsequent history of the case. The firm closure of such fistulæ is impossible without excision of the whole gangrenous area and the coaptation of healthy tissues. None of the intestinal coats around the fistula will hold a suture of any degree of tension. The intestinal peritoneum cannot be dragged over the openings, because it is rendered immovable by inflammatory adhesions, and is so friable that all sutures cut through. However, though these fistulæ cannot be firmly closed, yet the escape of feces from them can be deferred for a considerable time. And this is especially true if the patient has been prepared for laparatomy by a thorough emptying of the intestinal tract. When the fistulous opening cannot be closed by sutures, it may be covered by a large omental graft, extending around the intestine and reaching to the mesenteric attachment. Such a procedure will delay often for several days the escape of feces, and may result in cure without even the formation of a fistula.

There is a group of cases in which the gangrenous intestine and the fecal fistula are situated in a position not accessible for operation. This occurs in cases of pelvic suppuration with the history of purulent discharge from the anus. The fistula exists in the rectum, or in the sigmoid, which is often as inaccessible for operation as the rectum, on account of adhesion and contraction of the mesocolon. Under such circumstances all operations for closing the fistula are generally out of the question.

It is, however, fortunately situated in a position most favorable for spontaneous cure, being on the side of a straight portion of intestine, more or less immovable, and especially accessible to drainage and irrigation from above and to intestinal drainage and disinfection through the rectum. Indeed, in most cases, small rectal fistulæ—if properly treated—in no way interfere with the patient's chances of recovery, even though left unclosed.

To insure the spontaneous cure of fistulæ, it is important that there should be no obstruction in the distal portion of the intestine. All obstructing inflammatory tissue and bands of adhesion must be completely removed, and intestine adherent in sharp flexures must be straightened out. We thus obey a most important rule for the closure of all fistulæ by rendering the natural passage-way the easier one.

Of course, in all cases of gangrene of the intestine drainage is of the greatest importance, especially drainage by means of large straight tubes whenever we fear escape of feces. Care must be exercised in placing the drainage-tubes, that they do not exert any lateral or vertical pressure upon the diseased portion of intestine, as sloughing has occurred from this cause even in healthy structures.

Before concluding I will report briefly three cases of gangrene of the intestine, in which ideal surgical operations were impracticable, and which were cured by treatment such as I have been considering.

CASE I. *Abdominal and pelvic abscess, gangrenous intestine, entero-vaginal fistula.*—C. G., twenty-one years old, had a history of syphilis of two years' duration. Abdominal section was performed for double hydrosalpinx, and at this operation the peritoneum and the intestines were found perfectly normal with the exception of some healthy pelvic adhesions. Eight days subsequently it was necessary to reopen the abdomen on account of symptoms of intestinal obstruction, and because it was discovered that a free discharge of feces was taking place through the vagina. At the second operation the peritoneum was in the following condition: There was an abscess in the right inguinal region containing several ounces of pus. In immediate contact with this accumulation was a loop of small intestine, eighteen inches long. The whole loop was thickened, stiff, and friable. On the convex surface were two black, necrotic patches, two inches in diameter. A second collection of pus was found behind the left broad ligament. The sigmoid was in contact with this accumulation, and was firmly adherent to the posterior wall of the vagina. When separated from the vagina the bowel presented on its surface a necrotic patch, one and one-half inches in diameter. The fistulous opening through which feces had escaped into the vagina existed in the center of this patch, as there were no other portions of the intestine adherent to the vagina. The opening, however, was not patulous, and was obscured by the thickened diseased tissues around it.

As the patient was in a state of collapse, with an almost imperceptible pulse of 160, no radical procedure like resection or excision could be performed. The gangrenous spots were, therefore, left untouched. The small intestine was placed transversely across the abdomen, immediately above the upper angle of the incision. The sigmoid was freed from all adhesions and straightened as much as possible. And the abdomen was drained by means of three tubes through wh ch continued irrigation was conducted. The patient recovered perfectly without at any time having escape of feces by any but the natural passage. This is especially remarkable, because there was left unclosed a fistulous opening which had been large enough to permit the free escape of feces into the vagina before operation.

CASE II. *Abdominal abscess, gangrenous intestine.*—A. H. had been suffering for several years with pelvic pain, and recently with acute abdominal trouble with great pain, chills, hectic, uncontrollable nausea, diarrhea, and very free discharge of pus and blood by the rectum, and rapidly increasing emaciation. At the time of operation the abdomen was distended and tender, with marked fulnes and feeling of resistance, and dulness on percussion over the right side from the groin to above the umbilicus. Temperature, 103°, and pulse 130. Median laparatomy was performed and almost a quart of feculent pus was evacuated from an abscess cavity in the right side of the abdomen, which extended from the floor of the pelvis to two inches above the umbilicus, and inward to the median line. At the bottom of this cavity was found a pyosalpinx, which had been the cause of the trouble. Though a large mass of intestine walled in this collection of pus, yet there was but one gangrenous spot discovered. It was on a loop of ileum and was about two inches in diameter. No fistula was detected, though it is probable that a small valvular opening existed in this spot, as there had been a free purulent discharge from the bowel before operation. As the removal of the necrotic patch was impossible without resection, it was covered by a large omental graft. The intestine was placed in a straight line transversely across the abdomen, with the gangrenous portion at the upper angle of the incision; and two glass drainage-tubes were introduced, one to drain the healthy peritoneum and the other the site of the purulent accumulation.

Pulse after the operation was 160. On the fourth day feces escaped from the larger tube, viz., the one which drained the right inguinal region. The quantity increased daily until at the end of a week the total discharge of the bowel came through the abdominal incision. After this it gradually decreased, and six weeks after the operation the abdominal wound was firmly closed and all feces passed *per anum*. The woman is now in good health.

CASE III. *Gonorrheal pyosalpinx, with a fistulous opening into the rectum.* A. F. had been suffering with pelvic pain for three years. For several weeks before operation she had been unable to stand erect, and had frequent discharges of small quantities of pus from the anus. After each discharge the pain was temporarily relieved. Abdominal section was performed, and two pus tubes were removed. There was no abscess in the pelvis outside of the Fallopian tube. The right tube was adherent by its fimbriated end, low down on the anterior surface of the rectum. The discharge of pus had taken place at this point. Fecal odor was perceived after the removal of the tubes and irrigation of the pelvis. There was, however, no escape of feces until the glass drainage-tube was introduced, when a small quantity of dry feces was withdrawn from the tube by a syringe. The woman was unusually large and fat, and the fistula could not be brought into view, and was inaccessible for any attempt at closure. No such attempt was therefore made, reliance being placed altogether on the abdominal drainage-tube, and on drainage through a glass tube inserted in the anus. I felt especially confident in leaving the fistula patulous since the woman had been thoroughly purged for two days prior to the operation; and because it was in a position most favorable for spontaneous closure.

There was no discharge of feces from the abdominal drainage-tube until twenty-four hours after the operation, when the nurse, without direction, administered a turpentine enema, the result of which was intense pain throughout the lower half of the abdomen and the free escape of turpentine and water from the abdominal tube. For four days after this there was a feculent discharge from the abdomen, and on the fifth day a large quantity of flatus escaped through the abdominal tube while the rectal tube was removed for cleansing. The bowels were moved by glycerin enemata on the ninth day, everything coming by the natural passage. The patient was discharged cured, four weeks after the operation.

I have reported these cases because they illustrate the pathological condition to which I wish to call attention, and because they show what can be done without resort to the most heroic surgical operations. In conclusion, I will repeat what seems to me to be the most important points in the treatment of such cases:

1. Free purgation of the patient before operation.
2. Complete removal of all obstructing causes below the gangrenous intestine.
3. The adjustment of the gangrenous intestine in a straight line.
4. Thorough drainage and irrigation of the peritoneum.

---

FOURTH PAPER.

### SIX CASES OF LAPARATOMY.

BY JOHN H. PACKARD, M. D.,

Surgeon to the Pennsylvania Hospital.

THE clinical histories to which I shall ask the attention of the society this evening have seemed to me to present features of sufficient interest to warrant their being placed upon record. My own comments upon them will be very brief, but I shall be glad if the facts stated should elicit expressions of opinion on the part of others.

CASE I. was one in which the operation was exploratory. The patient, May O., *æt.* twenty-three, was admitted to the Pennsylvania Hospital October 16, 1889. Menstruation began at fourteen, and was always profuse and painful, often lasting ten days. She generally had leucorrhea between times, the discharge being sometimes thick and yellow. Married at eighteen, she had a difficult but not abnormal labor one year afterwards, getting up on the eleventh day and nursing the child until it was eighteen months old. Her menses came on about six weeks after her confinement, and have recurred regularly since.

Three years ago she began to feel pain in the right ovarian region, and detected a small swelling there. The pain and tenderness extended over most of the abdomen, but in less degree. There had been a steady growth in the tumor, which seemed to be about the size of a small orange. For about six months she had been failing in general health, losing over twenty pounds in weight. Her appetite was capricious, and her sleep much disturbed.

An exploratory laparatomy was decided upon, and performed October 21st. The abdomen being opened in the median line, the uterus and appendages were found to be normal, except that the left ovary was studded with small cysts. The tumor was found to be connected with the abdominal wall only, like a hemisphere attached by its flat side. The median opening was closed, the peritoneum being sewed separately with fine catgut. Next, the tumor was removed through an incision made parallel to Poupart's ligament. In dissecting it away, the peritoneum was again opened, and this wound was also sutured with fine catgut.

The patient reacted well from the ether; temperature in the evening, 100°. She complained of severe abdominal pain.

On the 22d the menstrual flow reappeared, and the pain passed away.

On the 30th, the ninth day, the dressings were changed, and both wounds were found perfectly united. She could now extend the right thigh completely, which had been impossible to her before the operation.

November 10th, the twentieth day, she was dressed and going about the ward.

On the 14th, she complained of pain, such as she had formerly had. This was thought to be symptomatic of a menstrual flow, but passed off apparently spontaneously.

She had no further symptoms, and left the hospital on November the 18th.

CASE II.—This patient, Fanny G., *æt.* twenty-seven, married, was admitted into the women's surgical ward of the Pennsylvania Hospital, October 3, 1889, at the request of Dr. L. F. Flick. She has had four children, the last one born two years ago. Her statement was, that four months previously she began to have bearing-down pains, especially when her bowels were moved; and there was a constant sense of something in the rectum which she desired to expel, and could not. On examination there was felt, just posterior to the os uteri, a very large, tense, uniform, fluctuating swelling. The woman looked very ill; her temperature, however, was only 101°.

I punctured the swelling in the median line, through the posterior wall of the vagina, and evacuated a small amount of blood, partly coagulated; a tube of soft rubber was introduced, but there was very little discharge through it, and it was retained with difficulty.

Five days afterward, there was a very free bloody flow from the vagina, supposed to be menstrual. She had severe pain in the hypogastric region, and frequent micturition, the urine containing pus. Evening temperature, 102°.

The next night there was a profuse hemorrhage, the origin of which was uncertain; it was checked by a tampon of iodoform-gauze and a T-bandage.

October 11th, the next day, a drainage-tube was again inserted into the cavity posterior to the uterus, and a tampon was kept in the vagina.

On the 12th the discharge became very offensive, and she had a good deal of abdominal pain, as well as nausea and vomiting. There was a considerable degree of fever, which lasted for days.

A solution of permanganate of potassium was used to wash out the supposed abscess-cavity, and by the 17th the discharge had ceased. I suspected that the case was one of extra-uterine pregnancy, and the same idea occurred to Dr. Goodell and to Dr. Joseph Price, both of whom examined the patient with me on occasions when they happened to be at the hospital.

On the 26th, another severe hemorrhage took place, and was checked as before.

On the 11th of November, the woman's condition being fairly good, she was etherized, and an abdominal section made. The mass behind the uterus was small, and there was a number of coils of intestine adherent to it; these were carefully separated. The left ovary and tube were normal. The right ovary was enlarged, and embraced by the fimbriated extremity of the distended tube; both were removed. Closure of the abdomen was effected by suturing the peritoneum separately with fine catgut, the muscular layer and skin with silkworm gut, and a few superficial stitches.

The discharge through the glass drainage-tube was, at first, pure blood, then bloody serum, and, finally, serum alone; the tube was removed on the third day.

Nothing further of note occurred in this case, and the patient left the hospital in good condition on the 9th of December, just four weeks after the operation.

Upon examination of the removed ovary it was found to have appended to it a sac containing a mass composed of loose meshes, like the villi of the chorion; under the microscope corresponding appearances were presented, and the case is therefore shown to be one of ectopic gestation. The minute embryo was probably carried away when there was a hemorrhage from the sac into the substance of the broad ligament, and escaped in the mass of blood evacuated from the hematocele.

CASE III.—Sarah Jane R., *æt.* thirty-four, colored, was brought into the Pennsylvania Hospital, December 4, 1889, in the evening, with a mass of small intestine upon the surface of the abdomen. Upon inquiry, she stated that in June last she was operated upon by Dr. Penrose for an abdominal tumor, the complete removal of which was impossible. She had had ever since then a sinus discharging on the front of the belly, and several large fibroids were distinctly to be felt within the cavity. For several months her general health had been failing, with loss of flesh, severe cough, and purulent expectoration.

On the evening of her admission to the hospital, she had a violent fit of coughing, and the cicatrix gave way, allowing the protrusion of the intestines. The exposed peritoneal surfaces were deeply congested, and the coils of bowel were lightly glued together with plastic lymph; the peristaltic movements were very plainly seen. The patient's condition was not one of shock, although the temperature was below the normal. There was no constriction of the protruded mass.

Between the time of her admission and my arrival at the hospital, towels wrung out of hot sublimate solution were kept constantly applied to the exposed bowel. The patient was then etherized, and the orifice in the abdominal wall carefully enlarged. The cicatrix seemed to be extraordinarily soft, giving way almost like wet paper. The bowels were attached rather firmly to the uterine tumors, which were of large size and irregular shape. In the manipulations necessary to returning the intestines, some of these adhesions were separated, and copious hemorrhage ensued from the surface of the tumors. This bleeding came from various points, some low down in the pelvis; and it was so free, and all attempts at ligation of the vessels failed so completely, that it seemed as if the woman must die on the table. Finally, by packing with iodoform-gauze the flow was checked; the bowels were carefully replaced; the abdominal wound was closed with large pins, and figure-of-8 turns of braided silk, and the usual dressings were applied.

On the 5th, there were no signs of bleeding, or of peritonitis, and the woman said she was very comfortable. She drank peptonized milk with a relish, and the only unfavorable symptom was a subnormal temperature.

In the evening, she vomited a little, and delirium came on, sometimes mild, but occasionally violent. At midnight she grew worse, her temperature fell still lower, and her pulse lost in force. Death occurred at 6 a m., about thirty-four hours from the time when the rupture of the cicatrix took place.

Perhaps I need hardly say that in this case the condition presented was a desperate one. But it seemed to me that, as interference was imperatively demanded, the proper course was to make it as effectual as possible, and hence that some examination of the cavity of the abdomen should be made before returning the protruded mass. The manipulations were conducted with extreme care and gentleness, and I think that if the opening had not been enlarged, the hemorrhage would have taken place just as certainly, but, of course, without the possibility of any measures being adopted to check it, unless a section had then been resorted to.

Case IV.—Minnie B., *æt.* thirty, was admitted into the Pennsylvania Hospital, December 9, 1889, at the request of Dr. J. H. Bradford. She has two children, aged ten and four years respectively. Ever since the birth of first she has had pain. Menstruation is very frequent and profuse. A mass about the size of an orange exists between the uterus and the rectum. The uterine cavity is readily felt, as if the organ were somewhat anteflexed.

This woman's general condition was bad; she was pale and thin; there was a double mitral murmur, and a very forcible apex beat. Her appetite was poor and she slept badly. There was a slight albuminuria.

After two weeks of general treatment, an operation for the removal of the ovaries was decided upon, and was performed, December 26th. It presented no unusual features, but there were some adhesions of the intestines to the appendages, as if there had at some time been a local peritonitis. These adhesions were easily broken up, and both ovaries and tubes were removed.

The day following the operation a severe cough came on, with very copious expectoration, moist râles all over the chest, and urgent symptoms of heart failure. This condition of things continued, in spite of energetic treatment, for three days. She had a high temperature every evening, was delirious and unmanageable, tearing her dressings off; yet the wound did well, and on the third day the glass drainage-tube was removed and a rubber one was inserted.

On the fourth day, January 1st, she got up and walked about the ward, and had to be strapped in bed.

January 3d she had a temperature of only 99°, and her cough is very much lessened; wound almost wholly united.

On the 15th, nineteen days after the operation, she was up and dressed, and although still pale and weak, was gaining steadily. A small sinus still remained where the drainage-tube had passed.

On the 1st of February, when I turned the wards over to my colleague, Dr. Ashhurst, this patient was ready to be discharged.

Case V.—Annie F., *æt.* twenty, was admitted into St. Joseph's Hospital, January 6, 1890, complaining of severe pain in the belly, most marked in the right iliac region, where there was great tenderness also. The whole abdomen was swollen, but at the part named there seemed to be more decided fulness and indistinct fluctuation. Her temperature was 104.2°; her pulse 130°; she lay on her back with her knees drawn up. The only history obtainable was that four days previously she had been seized with intense pain and vomiting, and that her bowels had been obstinately constipated from that time.

There were reasons for suspecting the existemce of salpingitis in this case.

On the 7th, the next day, the patient was etherized, and I performed an abdominal section. There was peritonitis, but not in an advanced stage; all the coils of intestine in the right iliac region were deeply congested, swollen and coated with flakes of lymph. The appendix vermiformis was thickened, stiffened, and so brittle that it came away in my fingers as I was examining it; its mucous lining was softened, and had a worm-eaten appearance, like that of the colon in a case of ulcerative dysentery. It had contained several white concretions, which escaped and were lost. A catgut ligature was tied firmly about the stump, which was then left to itself.

Both ovaries, with their thickened and tortuous tubes, were removed. The right one was about the size of a hen's egg, and contained a large mass of dark clot, constituting the chief part of its bulk; the left one was fibro-cystic.

After flushing the abdominal cavity with hot water, the wound was closed in the usual way, a glass drainage-tube being used, and the peritoneum being sutured separately with fine catgut. For the first twenty-four hours the patient was troubled with hiccough, which was relieved by ʒj doses of tincture of musk. On the evening of the day of the operation the temperature was 102.2°; the next evening 99°.

On the fourth day the drainage-tube was removed, and a cotton rope substituted for it. On the tenth day there was some gaping in part of the line of the wound, and a good deal of discharge with a somewhat offensive odor, not distinctly fecal, but suggestive of it; but this had disappeared on the following day. Balsam of Peru was now employed as a dressing, and by the twentieth day the healing was complete.

It should have been mentioned that on the third day after the operation the bowels were very freely moved, and thereafter there was no difficulty in this respect.

The patient has now been for over two weeks up and dressed every day, and only remained in the hospital from choice.

I would say in regard to what seems to me the most important feature in this case—the manner in which the appendix was dealt with—that the condition of the intestinal walls in the vicinity was such that I did not think they would bear any unnecessary handling, nor did I believe that sutures could be put in without risk of tearing. The certain and immediate danger attending a prolongation of the operation, and the slight prospect of benefit from a more elaborate procedure as to the appendix, determined me to take the chances of the simpler course.

CASE VI.—Mary J., *æt.* thirty-two, colored, single, was admitted to the Pennsylvania Hospital, January 18, 1890, on account of a uterine tumor, of which she had been conscious for about twelve months. She had previously noted that her menstrual flow had become much more copious and prolonged; her general health had failed, and she had lost flesh and strength.

On examination, the tumor, which could be distinctly felt through the abdominal wall, was found to occupy the posterior wall of the uterus, the cavity of which was three and a half inches in depth, and encroached upon by the mass.

Removal of the ovaries was decided upon, and performed on the 22d, five days after her admission. Both ovaries were cystic, and both tubes enlarged and tortuous. The uterine tumor was, as before described, one portion of it softened and bulging. Owing to the involvement of the broad ligaments, the uterus was too firmly fixed to admit of a hysterectomy. After the ligature, in a Staffordshire knot, had been tied on the left side, the tissues gave way, and there was very free bleeding, which was only controlled with great difficulty by catching the tissues in a large clamp, and including the whole mass in strong gut ligature.

The abdominal wound was closed and dressed in the usual way. At first only blood came from the drainage-tube, but by next day there was clear serum. The patient complained very little, but seemed drowsy and apathetic; her pulse ran up to 130°, but her temperature did not exceed 100°.

On the evening of the second day, as there was some nausea, small quantities of milk and lime water were given by the mouth, and m.v. of tincture of digitalis hypodermatically.

On the 24th her temperature was 101°; there was not much pain, but frequent gulping up of bile-colored liquid. She lay quietly most of the time. A rubber tube was substituted for the glass one.

On the 25th the condition was about the same, but the temperature had risen to 102°. The dressings were removed, and it was found that a knuckle of intestine had protruded; it was deeply congested, and adherent to the dressings by a layer of lymph. It was returned, and two new sutures applied. There was a very offensive vaginal discharge, apparently of decomposed blood.

On the 26th the temperature was 103°, the pulse almost imperceptible, and the breathing rapid and shallow. At about noon there was stercoraceous vomiting, and death took place at 1.30 P. M.

After death the wound was opened, and a good many adhesions, probably of various dates, were found; there was no general peritonitis. In Douglas's pouch there was a mass of blood resembling that discharged from the vagina, but no opening into that canal could be detected.

### DISCUSSION.

Dr. W. S. Forbes: It is refreshing to be reminded in such a clear and perspicuous manner as we have been to-night by our visitor from the west, Dr. McMurtry, that the great ribs of the vessel of sense holding our surgery of the peritoneum were announced in the beginning by our great countryman, McDowell—cleanliness, rapidity, drainage, and purgation. I doubt whether anything has since been added to materially strengthen this vessel. McDowell, in 1794, was a student of medicine in Edinburgh.

William Hewson, the friend and the pupil of John Hunter, had of late directed the attention of the medical mind to the anatomy and the physiological action of the lymphatic system of vessels, announcing the fact that the peritoneum was a great lymphatic space.

Hunter himself, while experimenting with cold applied to the comb of a cock, and telling the world the part was deprived of sensibility under such an application, directed his pupil, Physic, to carry on certain experiments concerning animal ligatures as he might suggest to him.

When McDowell returned to this country, he applied the knowledge he had acquired, and the result we have heard to-night. We have been told that the operation of ovariotomy, which McDowell did so much to advance, slumbered for nearly forty years after his death. The orator of the evening has not ventured to lay before us the cause of this prolonged slumber. Very properly, he may have thought this not a part of the history of ovariotomy. Nevertheless it may be instructive to investigate and lay bare the cause of this apparent cessation of interest in an operation which now commands so much attention. Let us hope Dr. McMurtry will visit us again, and in another paper place before us his views on this part of the subject.

I confess to the belief that abdominal surgery was held in abeyance during a long series of years by certain dogmas promulgated from medical centers lying east of the Appalachian range, and at variance with the principle McDowell taught and practised. These dogmas were at length forced to give way by the superior power of certain geniuses that live around us—notably the Atlees.

This is not, by any means, the first time in the history of medicine that dogmas have enslaved the medical mind. We are told that Hippocrates stated that cataract was opacity of the cystaline lens. Some centuries later Galen announced that this was not true. It was not until sixteen centuries had elapsed that Lasnier, in 1656, revived the truth of Hippocrates. We are told that only within half a century of Harvey's demonstration of the circulation of the blood, that Dr. Geynes, a Fellow of the Royal College of Physicians, was cited by a sheriff's writ to appear at the bar of the College and beg pardon for daring to differ from the dogma of Galen, and either recant or go to prison. We are told that on bended knee he did recant and beg Galen's pardon before the assembled College. It would thus appear that Galen ruled the medical mind with greater sway than his master, Marcus Aurelius, ever ruled the Roman world.

I think it will be found that for forty years dogmatic teachings held in abeyance all advance in peritoneal surgery.

The other papers read, one by Dr. Price, and the other by Dr. Penrose, very happily illustrate the principles enunciated by McDowell.

Dr. Morris, of New York: I came here in a receptive mood, and am not prepared to discuss the questions brought forward. In regard to drainage, I think that Dr. Price is correct, although I did not think so a year ago. It then seemed to me that even when the peritoneal cavity contained decomposing material, we should be able to dispose of it by means of drainage by way of the intestinal canal. I have since come to the conclusion that it is better to supplement this by artificial drainage in cases in which pus or other decomposing material exists in the peritoneal cavity. Otherwise I do not believe in drainage of the peritoneal cavity; never for the removal of serum, blood, or anything else but decomposing material. Experiments have shown that where the peritoneum is fairly normal, it is capable of removing large quantities of decomposing material.

Dr. Packard's error of diagnosis reminds me of an instance which occurred not long ago in my own practice. I had invited several friends to witness an operation on an interesting case of fibroid growth of the abdominal wall. At the operation I found that it was a gall-bladder filled with gall-stones. The clinical points which I obtained from this case I applied not long after in another case, and invited my friends to see another case of gall-bladder filled with gall-stones. I removed a large fibroid of the abdominal wall.

Dr. M. Price: Dr. McMurtry did not dwell upon the persecution side of the early history of ovariotomy. I can recall the time when to know Dr. Atlee was to know a scoundrel and a murderer in our profession. It was the same with others. These men suffered for years, but they are to be thanked through all time, beyond every other worker in the department of surgery, for their bravery.

I wish to refer to one case mentioned by Dr. Packard, that of a negro woman, where rupture of the cicatrix caused protrusion of the bowel. I call attention to this in connection with the open wound treatment of hernia. The McBurney treatment, which is now the rage in some circles, is simply absurd. It is nonsense to tell us that a scar is stronger than a well-healed wound. No one believes that scar tissue is stronger than natural tissues.

If I were asked what three things had done most to save life in abdominal surgery, I should reply, "Short ligature, drainage, and irrigation." I believe that more lives would be saved if a drainage-tube were used in every case. I think that I have never seen a case of septic and peritonitis except from omission of the drainage-tube. The tube must, however, be properly attended to; and until the surgeon can educate his nurse or himself to attend to a drainage-tube, *he should not open the peritoneum.*

Dr. George E. Shoemaker: If anything were needed to prove the value of drainage in abdominal surgery, we might take the cases reported to-night; they would prove its necessity in many cases, and its harmlessness in all. The risk from the drainage-tube itself is almost nothing if its accessories be kept clean, and for this purpose I consider the use of chemical antiseptic solutions necessary, even though they are omitted at the operation. There are occasions when one wishes to defer the change of dressing for a few hours, or a day, after accidental soiling. If a quantity of powdered boracic acid be applied round the tube, the serum which soils the dressing will be kept from decomposing better than by any other substance. A sponge should never be used to cover the mouth of the tube, but always absorbent cotton. The risk of fistula is not increased by a clean small tube, removed under forty-eight hours; and the strength of the cicatrix is not lessened by the time the patient is ready to be up.

Dr. G. Betton Massey: So safe are the methods of ovariotomy that the question at last arises when it should not be performed. That question has not been discussed on its merits. From an abundant evidence, I think it may be said that normal ovariotomy for pain is a failure. It may also be said that normal ovariotomy for nervous diseases, or Battey's operation, is a failure, unless the case is one of mere hysteria, and even then it will fail at times.

In regard to the removal of normal ovaries for fibroid tumors: This is a theoretical procedure, and can only be adopted as a permanent part of medical practice after its results have been demonstrated. I have at present several cases of fibroid tumor, which have mainly developed and continued to grow after the menopause, and am cognizant of many cases where this operation has been a complete failure in the arrest of hemorrhage.

The general practitioner can never form correct opinions on these subjects as long as the habit of presenting "wet specimens" to societies prevails, instead of waiting to report results. A glaring instance of this perversion was shown at the last meeting of this Society, when a specimen was shown several hours from the patient, to exhibit the triumph of oöphorectomy over electricity. That same case has been reported to-night by Dr. Packard as dying four days later; yet a record has been already made that is manifestly unjust.

Another question occurs to the general practitioner, and that is as to the treatment of tubal disease; or, in other words, catarrhal disease of the upper genital tract. In the treatment of catarrhal disease elsewhere, he does not call in the surgeon. During the last few years, the habit of taking out the tubes and ovaries for catarrhal disease has been greatly on the increase. It is my personal knowledge that many cases of catarrh of the tubes are operated on without any attempt to cure the condition by other treatment. I consider it an essential in the practice of medicine, that all conservative methods should be adopted before non-conservative procedures are resorted to.

Dr. J. M. Baldy: It is gratifying to know that the essential features of the operation of ovariotomy are to-day the same as those laid down by McDowell, with the possible exception of drainage. McDowell turned the patient on her stomach at the close of the operation, but made no permanent provision for drainage, except that he brought the ends of the ligature out of the lower angle of the wound. He probably did this more with the view of removing the ligature subsequently, than of favoring drainage. In my own mind there is no doubt of the importance of drainage. Dr. Morris says that he does not believe in drainage except where there are decomposing fluids. I once removed a fibroid tumor, and for eleven days there were two or three pints of serum discharged from the tube daily. This was not infection. The woman would have died without the drainage-tube. In one case of extra-uterine pregnancy I put in a drainage-tube, but in a few days removed it on account of fear of sepsis from the surroundings. Within three days I had to put the patient on the table and open the posterior *cul-de-sac* of the vagina, and remove three or four ounces of fluid which had not made the least progress toward absorption, and which was increasing in quantity. Such cases could be multiplied.

I do not question the ability of the peritoneum to take care of effused fluids, but often after an operation where there are many adhesions, there is no peritoneum left in the pelvis. The reports of post-mortems of the older ovariotomies plainly demonstrate that in many cases death was due to lack of drainage. It was quite common to read that three or four ounces of bloody serum were found in the pelvis at the post-mortem examination, and no other cause could be assigned for the death. To-day we get rid of this serum by drainage before death.

There are several points of great importance in Dr. Penrose's paper. Those dealing with pelvic masses frequently meet with dense adhesions to the intestines, where, in separating the adhesions, the coats of the bowels are torn down to the mucous coat, and, at times, even through this inner coat. At times we have a pelvic mass discharging through a fistula into the rectum. I have refused to operate in such a case, where the opening of the fistula was only a short distance within the anus. Dr. Penrose's experience would encourage me in future to operate in such a case. His dictum of free purgation for two or three days before operation, seems to be the safeguard in these cases against feces passing into the pelvic cavity, until it is shut off from the general abdominal cavity by plastic lymph.

That feces can be discharged through drainage-tubes with perfect impunity, is demonstrated by cases reported by Dr. Baer and others, where feces have passed for days, and the fistula subsequently closed spontaneously.

There is no question that many operations are done that should not be done, but I do not think that the criticism of Dr. Massey can hold good in the hands of men with well-recognized ability and experience.

In regard to the pelvic cases which are operated on without conservative treatment: If it is demonstrated that a given disease cannot be cured by a certain treatment, and that only the knife can effect a cure, there comes a time when it is folly to resort to these so-called conservative measures. It has been thoroughly demonstrated, in Philadelphia at least, that electrical treatment in pelvic inflammatory troubles is an absolute failure. There is not a single case that has been cured. The knife is the only thing that will cure some of these cases, and the only sensible thing is to use the knife at once, and not wait in each and every case.

Dr. B. F. Baer: Dr. Baldy has referred to a case of mine in which fistula followed ovariotomy. In this case, two days after the removal of double ovarian tumors, adherent to the colon, and necessitating considerable dissection, a drainage-tube was inserted, and the patient put to bed. Two days later the temperature was 102°, and there were tympanites. She was given a dose of Epsom salts, and the next morning the bowels moved, and the temperature became almost normal, but liquid fecal matter was flowing through the tube. I determined to try and find the fistula and close it, but, after working for an hour and a half, I failed to find it. I closed the abdomen with the expectation that the patient would die. She did pretty well for two days, when fecal matter again began to escape. I then treated it as an open wound, and the patient went home well twenty-eight days after the first operation. The lesson I learned from this case was to let fecal fistulas alone. I have had another case since, and I did not even irrigate. The patient went home in five weeks, perfectly well.

When I use a drainage-tube I always watch it carefully, and keep it emptied, and, in this connection, I may say that I think the little suction arrangement of Mr. Tait is a bad thing. I rarely have a drainage-tube in longer than forty-eight hours. The more experience I have, the less do I use a drainage-tube. In my last thirty-six cases I used the drainage-tube twice, and all these cases have recovered. I believe in drainage where it is necessary, but, in my experience, it is becoming less and less necessary. In general surgery, I think that drainage is used less than it was five years ago.

Dr. Massey: I am surprised to hear Dr. Baldy state that he does not know of a successful case of treatment of tubal disease, in view of several cases that have been reported to the Obstetrical Society. Electrical treatment has been followed by pregnancy in two or three instances in my practice.

Dr. John B. Roberts: In connection with one of the cases reported by Dr. Packard, I should like to put on record a rather curious case on which I operated a couple of weeks ago. I supposed that I probably had a carcinomatous disease of the transverse colon. I started to do an abdominal section, but, before I reached the peritoneum, cut into a cheesy mass, which was evidently tubercular, and which had not broken down. The cheesy matter was turned out, leaving a large cavity on each side of the middle line. Two drainage-tubes were put in, and the case is recovering rapidly. The tubercular collection was between the peritoneum and muscles, and felt, through the skin, like a hard, irregular mass, with a somewhat notched upper border.

Dr. Baldy: As illustrating the difficulty of diagnosis mentioned by Drs. Morris and Roberts, I can mention two cases. In one, where sarcomatous disease of the kidney was diagnosticated by two or three experts, the tumor turned out to be a fibroid or fibro-sarcoma of the abdominal wall. The second case was one of supposed cystic degeneration of a fibroid tumor. There were two lobes, one of which was supposed to have undergone suppuration. It opened spontaneously. The patient subsequently died, and the abscess was found to be in the abdominal wall. There was a hard nodule of tumor on the side of, and under suppurating cavity, which was adherent to the underlying tumor, and a large cystic mass on the other side.

Dr. McMurtry: It occurs to me to allude to one point in connection with the paper on drainage, and that is the antagonistic influence to drainage of the routine use of opium. I know that this point has been thoroughly discussed in Philadelphia, but those of us who practise elsewhere find, almost universally, when called to a case suitable for abdominal section, that the patient is under the influence of opium. This is a serious obstacle to the progress of the case. It masks the symptoms, dries up the secretions, prevents elimination, and does positive harm. If drainage is supplemented by purgation, it gives a more favorable aspect to the case, and promotes recovery.

My experience is the reverse to that of Dr. Baer. I find that the more abdominal work I do, the more frequently I use the drainage-tube.

Dr. Price: The statistics of the early operations in ovariotomy were not bad. It is to be noted that the results of exploratory and of incomplete operations were the same as now. The cases of exploratory operation recovered, while the incomplete operations were all failures. Of the twenty-one cases reported from 1809 to 1830, seven died and fourteen recovered. An interesting fact is that McDowell's fourth and fifth cases were negresses; in the one a cystoma, in the other a dermoid. It has been denied that these occur in the negress. The case of Smith serves to fortify what I have so often called attention to, and that is the injury to co-existing cystoma during labor. This has often occurred in my experience.

I am glad that Dr. McMurtry has called attention to the use of the decoction of senna. That case made a happy recovery. I value the preparation of the patient by the use of laxatives. It is difficult to distend a bowel that has been thoroughly evacuated.

Schroeder, that great and just ovariotomist, entirely concedes the honor of ovariotomy to America. He says: "McDowell, of Kentucky, however, was the first man to perform ovariotomy in a rational and deliberate manner."

In regard to drainage: After the removal of healthy tumors drainage is not necessary; but where, as Dr. Baldy has stated, the peritoneum is stripped from the pelvic basin, drainage becomes essential, and in pus cases it cannot be dispensed with.

Nerve specialists and electricians talk about a class of cases with which we are not really dealing, except as they are placed in our hands by these specialists; they put into our hands a class of cases which we hesitate to touch.

In regard to the removal of appendages for fibroid and small myomas, it has been one of the most successful operations ever devised. In Mr. Tait's first series, completely reported, the mortality was only two per cent., and this is explained by accidental causes,—the existence of peritonitis and other vicious complications. In about eighty-five per cent. the menopause was completely established.

Dr. Penrose has, this evening, shot over the heads of the surgical profession. It will be a long time before the wisdom of this council is fully appreciated. Surgeons will have some sad and distressing experiences before they adopt the procedures suggested.

---

THE dangers of antipyrin are now being descanted upon by the daily papers, inspired thereto by the reckless home-dosing which has been carried on during the last few weeks. Antipyrin is not so dangerous as quinine, camphor, whiskey, cherry pectoral, chloral, or opiates—drugs that are freely used at all times by the laity.—*Times and Register.*

